# Adsorption-Enhanced Ceramic Membrane Filtration Using Fenton Oxidation for Advanced Treatment of Refinery Wastewater: Treatment Efficiency and Membrane-Fouling Control

**DOI:** 10.3390/membranes11090651

**Published:** 2021-08-25

**Authors:** Haotian Mu, Qi Qiu, Renzhen Cheng, Liping Qiu, Kang Xie, Mingchang Gao, Guicai Liu

**Affiliations:** 1 School of Civil Engineering and Architecture, University of Jinan, Jinan 250022, China; muhaotian1997@163.com (H.M.); 18366105822@163.com (R.C.); cea_xiek@ujn.edu.cn (K.X.); gaomc555@163.com (M.G.); 2 School of Water Conservancy and Environment, University of Jinan, Jinan 250022, China; qiuqi_1994@163.com; 3 Research Center for Functional Material & Water Purification Engineering of Shandong Province, Jinan 250022, China

**Keywords:** refinery wastewater, ceramic membrane, combined process, membrane-fouling model, membrane-fouling control

## Abstract

With the development of the refining industry, the treatment of refinery wastewater has become an urgent problem. In this study, a ceramic membrane (CM) was combined with Fenton-activated carbon (AC) adsorption to dispose of refinery wastewater. The effect of the combined process was analyzed using excitation–emission matrix (EEM), ultraviolet-visible (UV-vis) and Fourier transform infrared spectroscopies (FTIR). Compared with direct filtration, the combined process could significantly improve the removal of organic pollution, where the removal rate of the COD and TOC could be 70% and the turbidity removal rate was above 97%. It was found that the effluent could meet the local standards. In this study, the membrane fouling was analyzed for the impact of the pretreatment on the membrane direction. The results showed that Fenton-AC absorption could effectively alleviate membrane fouling. The optimal critical flux of the combined process was increased from 60 to 82 L/(m^2^·h) compared with direct filtration. After running for about 20 d, the flux remained at about 55 L/(m^2^·h) and the membrane-fouling resistance was only 1.2 × 10^12^ m^−1^. The Hermia model revealed that cake filtration was present in the early stages of the combined process. These results could be of great use in improving the treatment efficiency and operation cycle of refinery wastewater.

## 1. Introduction

With the development of the refining industry, the threat to the environment caused by pollutants [1,2] contained in refinery wastewater has attracted more and more attention [3]. It is difficult for conventional treatments [4,5] to remove pollutants in wastewater such that the local standards are met; therefore, deep treatment processes are essential. Membrane filtration [6,7,8], activated carbon (AC) adsorption [9,10,11] and advanced oxidation processes (AOPs) [12,13,14] have a wide range of applications in deep treatment processes of refinery wastewater due to their unique advantages. However, the treatment efficiency of a single technology is limited [15]. Based on these, it is necessary to design suitable combined processes for the treatment of refinery wastewater. 

Compared with a single process, a combined process can remove pollutants in wastewater more effectively and make full use of the advantages of various methods and make up for their shortcomings [16]. After several years of research, some processes [17,18,19,20,21,22,23,24] were designed and researched for the treatment of refinery wastewater. According to the previous research, a combined process that consists of an AOP and AC absorption displayed a great effect in the treatment of wastewater [25,26]. An AOP has a strong capacity to degrade refractory macromolecular substances into small molecular substances with a fast reaction speed. AC adsorption could be used to remove the pollutants at very low concentration levels in wastewater [27]. Based on these methods, the combined process of AOPs and AC adsorption has great potential in the treatment of refinery wastewater. However, the application of AOPs and AC would introduce new colloidal substances into the water after processing, and these substances are difficult to separate. To solve this issue, a membrane was introduced. 

Membrane separation is one of the most commonly used methods for the deep treatment of refinery wastewater due to its many advantages [28,29,30,31,32]. Through previous research, combining other processes with a membrane in the treatment of refinery wastewater is an effective method for improving the quality of effluent and output [33,34,35]. However, membrane fouling is the main reason that restricts the application of membranes in wastewater treatment [36]; therefore, controlling membrane fouling is the key to improving the efficiency of membrane filtration [28,32,37,38]. It was found from previous studies [39] that pollutants in wastewater could be removed effectively via pretreatment so that the operating pressure of the subsequent membrane system would be decreased and then membrane fouling was alleviated. Among the possible membranes, it is feasible to combine a ceramic membrane (CM) with an AOP and AC adsorption to improve treatment efficiency and reduce membrane fouling [40,41,42,43,44,45]. The simultaneous combination of CM with an AOP and AC adsorption is rarely applied in the deep treatment of refinery wastewater; as such, the synergistic effect and membrane-fouling behavior are still unclear, which need further research and verification.

In this study, Fenton oxidation, AC adsorption and CM filtration were selected as the main components of a combined process for the deep treatment of refinery wastewater. Single-membrane filtration and a combined process were simultaneously operated to compare and analyze the treatment efficiency and membrane fouling. The model of membrane fouling was built for revealing the mechanism of membrane fouling. This study aimed to provide new insights to improve the efficiency of membrane processes for treating refinery wastewater and alleviate membrane fouling.

## 2. Material and Methods

### 2.1. Material and Reagents

In this study, the CM was provided by the Advanced Ceramic Research Institute of the Zibo High-tech Industrial Development Zone. Its structure is shown in Figure S1. There are channels inside the framework of the membrane module. The feed entered the internal channel through the pores on the membrane’s surface and moved to the low-pressure area in the channel due to the suction from the pump. The liquid flowed out of the membrane module assembly through its water outlet and was finally collected. The size was 240 mm × 250 mm × 6 mm, the pore size was 100 nm and the mechanical strength was ≥15 MPa. The pure water flux could reach 500 L/(m^2^·h) under a pressure of 0.03 MPa, which showed a good permeability.

The adsorbent was granular activated carbon with a porosity of >70. Fe(NH_4_)_2_(SO_4_)_2_·6H_2_O and H_2_O_2_ were the ARs used for the Fenton oxidation and K_2_Cr_2_O_7_ used for the analysis of COD was GR. The water used in the experiment was taken from an oil refinery in Shandong. 

The feed was from the secondary biochemical effluent of a refinery, where the turbidity of the feed was about 1.78 NTU and the COD was 36~40 mg/L.

### 2.2. The Design of the Experiment

#### 2.2.1. Direct CM Filtration Experiment

The CM was directly used to treat the refinery wastewater and was operated under the condition of constant pressure filtration. The effluent was collected in a tank to test its quality for analysis of the treatment effect. During the operation, the critical flux of the CM was measured using the flow ladder method and the speed of the pump was controlled to adjust the pressure on both sides of the membrane. The flux was changed every 30 min, and the data of the flux and *TMP* was recorded every 5 min. Three initial fluxes of 30, 60 and 90 L/(m^2^·h) during the operation of the filtration were selected and the variation of the flux and *TMP* was noted to analyze the membrane-fouling behavior.

#### 2.2.2. Experimental Setup of the Combined Process

**Figure 1:**
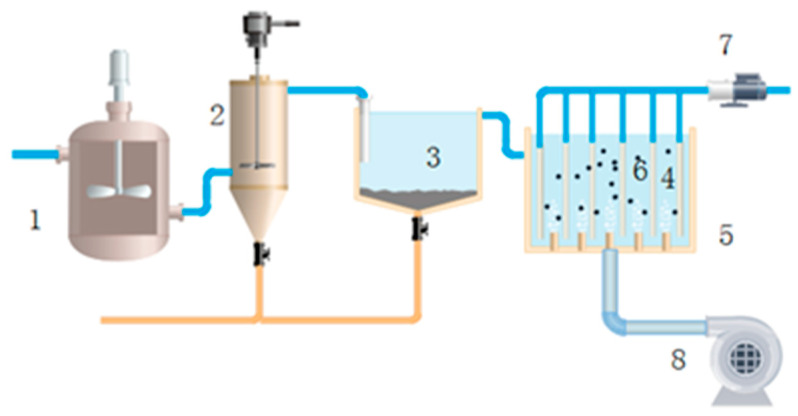
Schematic diagram of the combined process: 1. regulating tank; 2. Fenton reaction tank; 3. sedimentation tank; 4. ceramic membrane; 5. membrane pool; 6. activated carbon powder; 7. production water pump; 8. blower.

The experimental setup of the combined process is shown in Figure 1. The feed first entered into a regulating tank equipped with a stirring blade, acid addition equipment and a real-time online pH control device, which was used to adjust the pH of the wastewater to suitable conditions for Fenton oxidation. The effluent stayed for 5 min, became an outflow and entered into the Fenton reaction tank. The tank was equipped with agitating equipment that maintained 120 r/min to increase the reaction efficiency. The dosage of the regents was controlled by metering pumps. The pH condition was controlled at 4.1 and the retention time was 30 min. After the oxidation, the water’s pH was adjusted to 7 by adding NaOH in the pipe between the Fenton reaction tank and the sedimentation tank, and the water entered the sedimentation tank for precipitating the sludge effectively. The water stayed in the tank for 45 min and then flowed into the membrane pool in which the activated carbon was directly added to the membrane tank and the concentration was 40 g/L. After running for 12 d, the activated carbon was changed at a carbon change rate of 4%/d. Continuous aeration at the bottom of the pool ensured that the activated carbon was suspended in the water, and agitation was used on the surface of the ceramic membrane to reduce the deposition of activated carbon and pollutants on the membrane’s surface. Finally, the water was filtered using the CM and collected in a tank for the analysis of the treatment efficiency, where the run time was about 40 min. The changes in flux and *TMP* were noted every day to study the membrane-fouling behavior.

### 2.3. The Method of Analysis

The COD and TOC in the feed were analyzed using the potassium dichromate method and total organic carbon analysis (multi N/C^®^ 3100, Jena, Germany), respectively. The turbidity and chromaticity were measured using a turbidity chromaticity meter (2100AN, Hach, CO, USA). The conductivity and pH were determined using a conductivity analyzer (DDS-307, Lei Ci, Shanghai, China) and a pH meter (S220, Sedrwas, Goettingen, Germany), respectively. A dual-beam UV-visible spectrophotometer (TU-1901, General Analysis of Beijing, China) was used to scan and analyze the pollutants in the feed. The wavelength range was 190~600 nm, the scanning step was 0.25 nm and the optical path of the quartz colorimeter was 10 mm. FTIR (NICOLET WAS10, Seymour, Waltham, MA, USA) was used to analyze the pollutant categories and organic functional groups in each sample. The resolution was 4 cm^−1^, the number of scans was 32 and the scanning interval was 4000~550 cm^−1^.

A three-dimensional fluorescence spectrum (EEM) was determined using a synchronous absorption three-dimensional fluorescence spectrometer (HORIBA Aqualog^®^, British HORIBA) for the qualitative analysis of each water sample. The excitation spectra (EX) were scanned from 200 to 450 nm and the emission spectra (EM) were scanned from 240 to 600 nm. The scanning velocity was 50 nm/s.

### 2.4. Membrane-Fouling Analysis

In the experiment, the membrane pressure on both sides was adjusted according to the speed of the pump and the flux of clear water collected during a certain period was calculated using a stopwatch and a measuring cylinder. The flux was calculated under different pressures according to the following Equation (1):

(1) $$J=\frac{V}{tS}$$

where *J* was the permeate flux (L/(m^2^·h)), *V* was the filtration volume (L), *S* was the membrane area (m^2^) and *t* was the filtration time (h).

The inherent resistance of the CM was a basic indicator to measure the membrane performance. The *TMP* and flux were used as calculation parameters of the inherent resistance. Equation (2) was used for this calculation:

(2) $$R_{m}=\frac{TMP}{\mu J_{0}}$$

where *R_m_* was the intrinsic membrane resistance, the value of *TMP* was equal to the membrane pressure on both sides, *J*_0_ was pure water flux (m^3^/(m^2^·s)) and *μ* was assumed to be the viscosity of water at 25 °C (0.8937 × 10^−3^ N·s/m^2^).

Based on Darcy’s law, the membrane-fouling resistance was calculated:

(3) $$J=\frac{TMP}{\mu \;\left(R_{m}+R_{f}\right)}$$

*R* = *R_m_* + *R_f_* (4)

where *R* was the total membrane resistance (m^−1^) and *R_f_* was the membrane-fouling resistance (m^−1^).

The critical flux was an important indicator for analyzing the rate of membrane fouling. In the experiment, the critical flux of membrane filtration was measured using the flow ladder method. The flux was changed every 30 min, and the flux and *TMP* were recorded every 5 min. The critical flux was determined by analyzing the *TMP* and flux.

### 2.5. Membrane-Fouling Model

The Hermia model was used to fit the membrane-fouling model during the experiment and analyze the type of membrane fouling. The Hermia model contains four types: complete blockage, intermediate blockage, standard blockage and cake filtration. Complete blockage, intermediate blockage and standard blockage can be classified into a block filter and are produced by membrane pore blocking. The cake filtration model is based on the screening effect that particle pollutants carry via a filtrate cake that is formed on the membrane surface. The thickness of the cake and resistance increased with the running time. The specific equation is shown in Table 1.

## 3. Results and Discussion

Separate membrane filtration and combined processes were adopted to treat the feed. By analyzing the removal effect of pollutants in the wastewater and membrane-fouling behavior, the treatment mechanism of the combined process and the reason for the membrane fouling were obtained.

### 3.1. Treatment Efficiency

#### 3.1.1. The Removal of Organic Pollutants and the Turbidity

**Figure 2:**
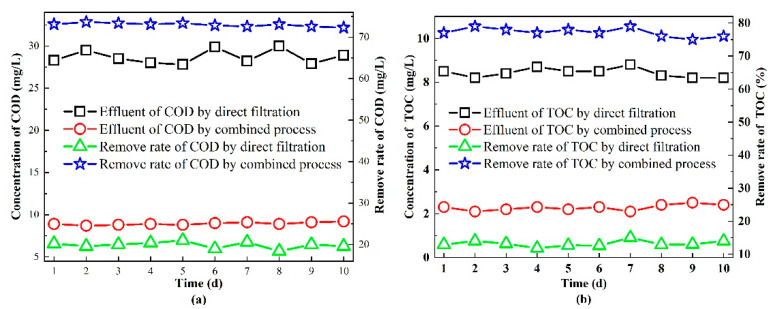

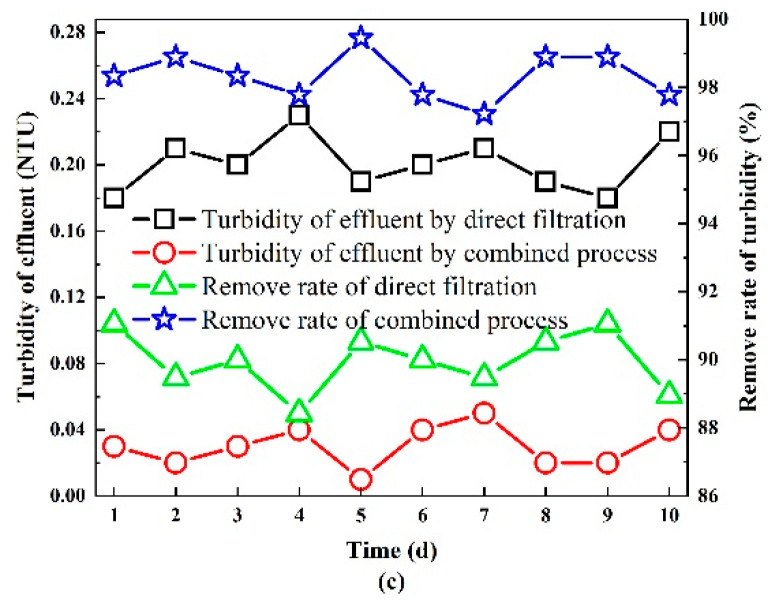
Removal of organic pollutants and turbidity using direct filtration and combined processes: (**a**) the removal of TOC from the refinery wastewater; (**b**) the removal of COD from the refinery wastewater; (**c**) the removal of turbidity from the refinery wastewater.

The removal effect of organic pollutants in the refinery wastewater can be reflected using the TOC and COD. Under the condition of direct filtration, the removal rate of the COD and TOC was about 20% and 14%, respectively, and the concentrations of the COD and TOC were 28~30 mg/L and about 8.5 mg/L in the effluent, respectively (as shown in Figure 2). It was found that the removal rate of organisms using direct membrane filtration was stable but relatively low. This might have been because the organics in refinery wastewater were mostly dissolved [28]. After the filtration, the suspended particles and oil with small grains were retained but a large number of organics could go through the pores, which resulted in the low removal rate observed.

Compared with direct filtration, although the treatment effect of the organics using the combined process fluctuated slightly, the removal rate of the COD and TOC remained above 70% under the combined process. The system operated stably and the COD and TOC in the effluent were lower than 10 mg/L and 2~3 mg/L, respectively. The result met the quality requirements. It was speculated that the process of Fenton oxidation and AC adsorption could effectively improve the removal effect of CM on organic pollutants in refinery wastewater.

The turbidity in the refinery wastewater was high; therefore, it should be further analyzed. The turbidity removal effect is shown in Figure 2c. It can be seen that the direct filtration using the CM had a good effect on removing the turbidity, where the removal rate could reach about 90%. The turbidity in the feed could be reduced from 1.9 to 0.2 (NTU) after the filtration and the effluent could meet the local standard. The result showed that the concentration of the turbidity in the refinery wastewater could be effectively reduced by the direct filtration of the CM. Some research [41] revealed the treatment effect of CM on wastewater with different turbidity levels. It was indicated that more than 98% of the turbidity was removed during the filtration of CM under all the tested conditions. Meanwhile, the removal effect of turbidity using the combined system showed that the turbidity removal rate was above 97% and the turbidity in the effluent was lower than 0.05 NTU under the tested conditions. Therefore, compared with direct filtration, the removal of turbidity could be further improved using the combined process.

#### 3.1.2. Fluorescence EEM Spectra

**Figure 3:**
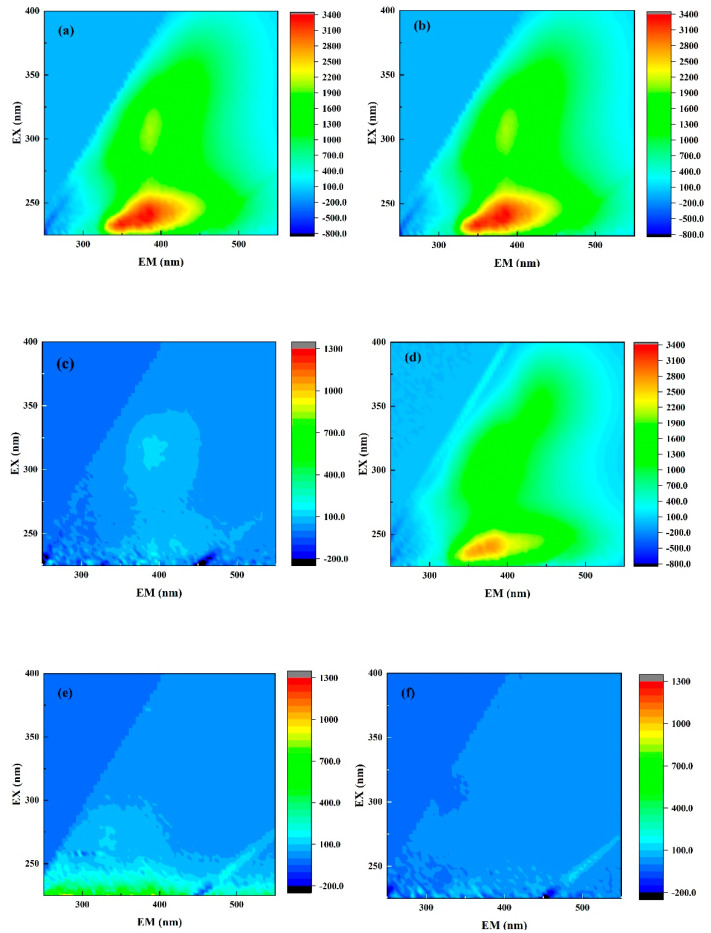
EEM diagram of water: (**a**) feed; (**b**) single TCM; (**c**) after Fenton oxidation; (**d**) dosing Fe^2+^ for coagulation; (**e**) absorbed by AC; (**f**) effluent. The excitation spectra (EX) were scanned from 200 to 450 nm and the emission spectra (EM) were scanned from 240 to 600 nm. The scanning velocity was 50 nm/s.

EEM was widely used as a useful method for the analysis of pollutant composition in water [46,47,48,49,50]. In this study, EEM was used to analyze the composition in the refinery wastewater at different stages. It was found that the EEM spectra could be divided into two regions: λ_EX_/λ_EM_ = 245 nm/385 nm and λ_EX_/λ_EM_ = 305 nm/385 nm (as shown in Figure 3a). According to previous studies [51,52], the peaks might be related to alkanes and polycyclic aromatic hydrocarbons, such as tyrosine and tryptophan. As shown in Figure 3b, the peak intensities of the two regions showed no obvious change. It was noted that most organic matter, such as aromatic compounds, was rarely intercepted using direct membrane filtration. The organic pollutants in the refinery wastewater could be significantly reduced using Fenton oxidation (shown from Figure 3c), where the peak removal rates at 245 nm/385 nm and 305 nm/385 nm were above 95% and 94%, respectively. The locations of the peaks drifted, which meant that macromolecular substances, such as polycyclic aromatic hydrocarbons, turned into substances with a lower molecular weight, such as fulvic acid. To further explore the removal mechanism, the removal effect was researched via dosing with PFS for flocculation alone (as shown in Figure 3d). The result indicated that the removal of fluorescent substances in the refinery wastewater using the Fenton system was a synergistic process of flocculation and oxidation, and the removal rate was relatively low when using flocculation alone, which indicated that the oxidation played a leading role. The area of the peak was further decreased after the AC absorption (as shown in Figure 3e), where the peak was below 50 and the removal rate was above 96% after the absorption. It was supposed that some small molecules, such as fulvic acid and humic acid, were thoroughly adsorbed on the surface or in the pores of AC during the process [53]. After the pretreatment of Fenton oxidation and AC absorption, the water was filtrated using CM and, as a result, the pollutants in the feed were completely removed using the combined process (as shown in Figure 3f). Therefore, compared with the result of direct filtration, the treatment effect of refinery wastewater using the combined process was better.

#### 3.1.3. The UV-vis and FTIR Analyses

**Figure 4:**
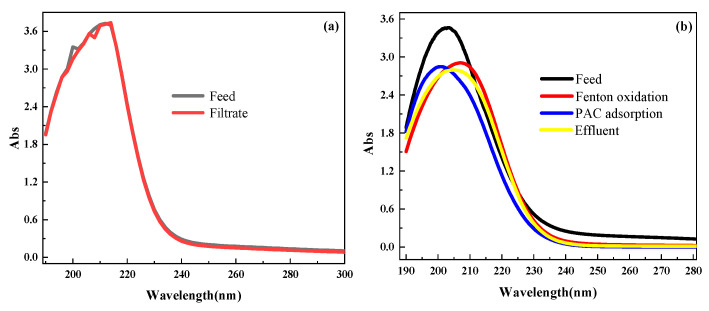
The changes in UV-vis for varied effluents: (**a**) the changes in UV-vis during the direct filtration using the ceramic membrane; (**b**) the changes of UV-vis in each phase of the combined process. The wavelength range was 190~600 nm, the scanning step was 0.25 nm and the optical path of the quartz colorimeter was 10 mm.

UV-vis is an important method for the analysis of dissolved organic matter in wastewater. The method was used in many studies to analyze the concentration of COD in wastewater and the transformation of organic matter [54,55]. As shown in Figure 4, the change of organics in the refinery wastewater after filtration was not obvious from the UV-vis, indicating that direct membrane filtration was not ideal for removing the pollutants in the refinery wastewater. After the Fenton oxidization, the absorbance for wavelengths ranging from 190 to 230 nm was effectively reduced, while the location of the peak at 220 nm changed a little and the peak at 202 nm was red-shifted to 210 nm. This indicated that aromatic compounds could be effectively reduced using Fenton oxidation. It can be seen from Figure 4 that the absorbance (Abs) values at all wavelengths decreased after the absorption by the AC, indicating that the small pollutants produced after the Fenton oxidation could be removed by the AC. In the end, the absorbance of the effluent treated using the combined process was significantly reduced compared with the direct membrane filtration, which indicates that the pretreatment of Fenton oxidation and AC adsorption could promote the removal effect of the CM for organics in the refinery wastewater.

**Figure 5:**
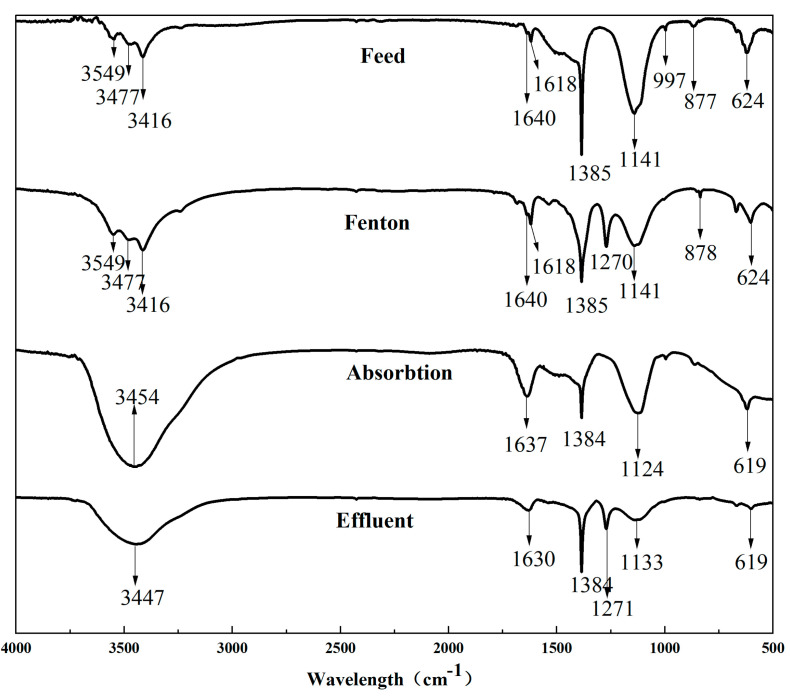
The changes in FTIR for varied effluents: the resolution was 4 cm^−1^, the number of scans was 32 and the scanning interval was 4000–550 cm^−1^.

FTIR is an effective method for analyzing the categories of pollutants and functional groups of organic compounds in water [56]. Through FTIR analysis, pollutants in water can be effectively identified and the rules of transformation about organic compounds under different treatment conditions can be analyzed. The feed and effluent of the different stages, including Fenton oxidation, AC absorption and the membrane, were each analyzed using FTIR (Figure 5). As shown in Figure 5, after the Fenton oxidation, the peak in the range from 1640 cm^−1^ to 1141 cm^−1^ decreased, which indicated that the Fenton oxidization could effectively reduce the concentration of sulfonic acid ester, amines and aromatic pollutants, and the result was consistent with the results of the EEM, where the peaks at 1384 cm^−1^ and 1141 cm^−1^ were also significantly lower compared with the feed. We found that a good treatment effect on sulfate lipids and amines could be achieved by Fenton oxidation. The peaks at 1640 cm^−1^ and 3416 cm^−1^ significantly decreased after the AC absorption, where fulvic acid and other small molecule substances in the refinery wastewater could be removed. Moreover, the concentration of amine compounds in the effluent was further reduced by the combined process of Fenton oxidation and AC absorption. However, after oxidation, a new peak appeared at 1270 cm^−1^, which might have been the anti-symmetric stretching vibration of the SO_3_ of the sulfate RO-SO_2_-O-. After the analysis, it was determined that the R groups in R_1_O-SO_2_-OR_2_ in the sulfate were oxidized to form the sulfate, and the sulfate was difficult to completely remove using the process of AC adsorption. It can be seen from Figure 5 that the sulfate was mainly removed using CM filtration. It was confirmed from the FTIR result that the molecular substances, such as aromatic pollutants, could be decomposed to low-molecular-weight organic matter and inorganic matter, which was further removed using AC absorption and CM filtration, and the treatment effect of the combined process was better than the direct membrane filtration.

### 3.2. Membrane-Fouling Behavior

#### 3.2.1. Critical Flux

The proper critical flux can ensure the stable operation of the membrane system. In this study, the critical flux was used to measure the operating efficiency [57] and reflect membrane fouling.

**Figure 6:**
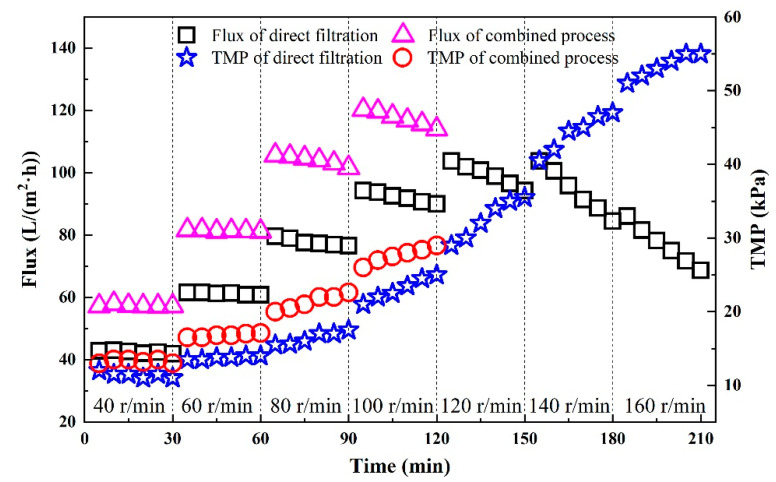
The changes in the critical flux and TMP: the critical flux of the membrane filtration was measured using the flow ladder method. The flux was changed every 30 min, and the flux and *TMP* difference in this period were recorded every 5 min.

The TMP was gradually improved by changing the speed of the pump to confirm the critical flux in the operations of the direct filtration and the combined process. The result is shown in Figure 6. During the operation of direct filtration, the flux and *TMP* both increased when the speed of the pump increased. In the initial stage of the operation, they remained relatively stable after being changed. When the speed increased to 80 r/min, the flux had a small downtrend (80 to 76 L/(m^2^·h)) within a short time. As the speed increased, the downtrend became more and more obvious with the faster growth of the TMP. It was seen that the flux increased first and then decreased with the continuous increase in *TMP* after the speed exceeded 60 r/min. According to the definition in [58], it was indicated that when the CM was used alone for filtration, the critical flux was 60 L/(m^2^·h) and the corresponding *TMP* was 15 kPa.

As for the combined process (shown in Figure 6), when the *TMP* was 14 kPa, the critical flux could reach 57 L/(m^2^·h) and the flux and *TMP* were relatively stable. When the *TMP* increased to 16 kPa with the increase in speed, the critical flux reached 82 L/(m^2^·h). As the speed continued to increase, the *TMP* began to rise within a short time and the flux decreased gradually. Therefore, the critical flux of the CM in the combined process was 82 L/(m^2^·h) and the corresponding *TMP* was 16 kPa.

Compared with the direct filtration, the critical flux during the combined process was increased by about 37%, which meant that a greater water yield was created using the combined process. The reason for this might have been that the large molecules of organic matter in the wastewater were decomposed by the pretreatment of Fenton adsorption into small molecules, such as sulfate. These were more likely to pass through the membrane pores, which could effectively alleviate membrane fouling. Therefore, the pretreatment of Fenton absorption improved the flux, reduced the pollution in the water and effectively slowed membrane fouling.

#### 3.2.2. Flux Decline and Membrane Resistance

During operation, membrane fouling can appear due to concentration polarization, cake layering and pore blocking [59], and it is inevitable. In this study, the reason for the membrane-fouling behavior during operation was further analyzed and the influences of pretreatment for membrane filtration were researched.

**Figure 7:**
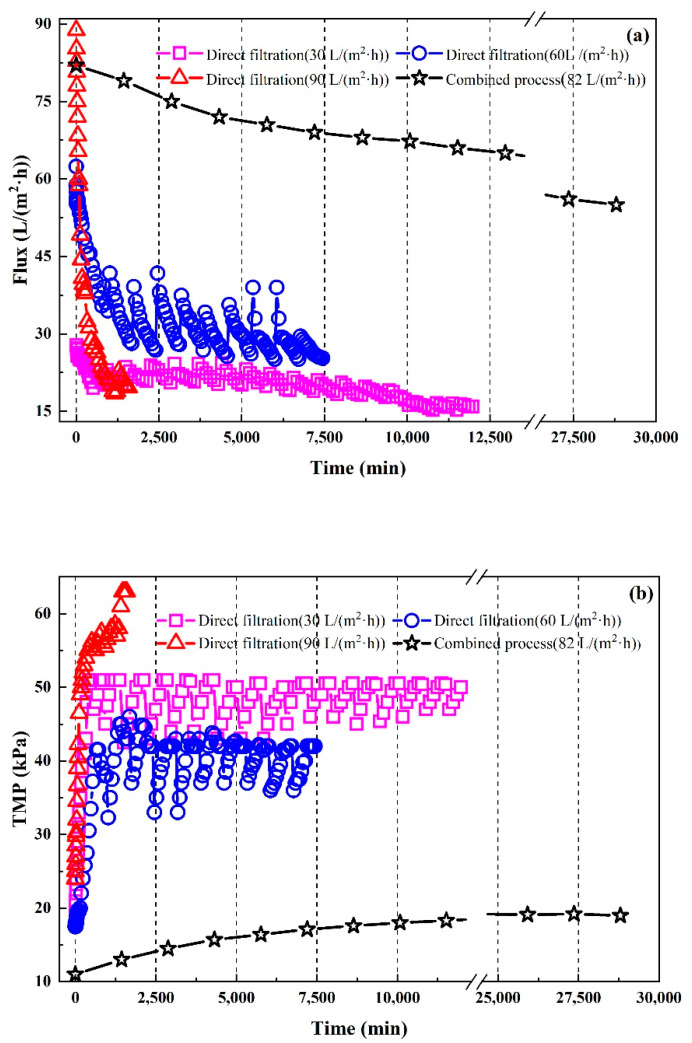
Changes in the flux and *TMP* of the ceramic membrane: (**a**) flux changes during the membrane filtration and combined processes with different initial fluxes; (**b**) changes in the *TMP* in the membrane filtration and combined processes with different initial fluxes: during the process of direct filtration, the data was obtained by adjusting the peristaltic pump speed (60, 80 or 140 r/min) to adjust the bilateral membrane pressure and a stopwatch and measuring cylinder were used to calculate the flux of water passing through the membrane during a certain time, where the flux was calculated using Equation (1). The initial flux of the combined process was selected according to Section 3.2.1 and the calculation method during the process was the same as for direct filtration.

In order to further analyze the membrane-fouling behavior during direct filtration, the changes in flux and *TMP* under different conditions of initial flux (Figure 7) were analyzed. As shown in Figure 7a,b, when the initial flux was 30 L/(m^2^·h), the flux of the CM and the *TMP* changed rapidly. The flux decreased from 30 to 18 L/(m^2^·h) during the first 500 min of operation and the *TMP* increased from 17 to 51 kPa. Therefore, the CM was polluted rapidly during this time and the same phenomenon occurred in the conditions where the initial fluxes of 60 and 90 L/(m^2^·h) were used. When the initial flux was higher, the decrease in flux was more obvious. During the operation, intermittent operation mode (operating for 12 h and intermission for 12 h) was adopted, where the flux increased and the *TMP* decreased synchronously after the intermission.

The observed phenomena might have been caused by cake filtration and concentration polarization on the membrane’s surface in the initial phase of operation. When membrane fouling was mainly a cake layer, intermittent operation mode could reduce the accumulation of cake on the membrane surface and improve the operating flux. With the extension of the running time, the change in the flux and *TMP* tended toward being stable. This might have been because the membrane fouling was dominated by membrane hole obstruction. When the reason for the membrane fouling was membrane hole obstruction, the intermittent operation mode had little influence on the *TMP* and flux. When the initial flux was 60 L/(m^2^·h), the final flux fluctuated in the range from 25 to 30 L/(m^2^·h) and the *TMP* varied from 45 to 55 kPa, which was higher than in the other condition. This result shows that the membrane fouling was controlled the best under these conditions. This was because the initial flux of 60 L/(m^2^·h) was the critical flux of the CM [60].

For the combined process, the changes in the flux and *TMP* are shown in Figure 7a,b. It can be seen that in the first 5 days, the flux decreased rapidly but the *TMP* increased relatively slowly, where it was only 6 kPa higher than the initial operation pressure. Eventually, the *TMP* stabilized at about 19 kPa. At the later stage of operation, the flux decline also slowed down gradually. After 20 days, the flux remained at about 55 L/(m^2^·h). By comparing this with the result of the FTIR, membrane fouling was further analyzed. According to the analysis, we concluded that Fenton oxidation could effectively reduce the amount of aromatic compounds in the water, which were considered to be the main reason for the membrane fouling [61]. These were removed or oxidized into molecules with smaller molecular weights [62,63,64]. Therefore, compared with direct filtration, the combined process could slow down the phenomenon of flux decline and extended the operating cycle.

To further analyze the flux and *TMP* under three different initial fluxes (30, 60 and 90 L/(m^2^·h)), the membrane resistance under three working conditions was analyzed (Figure S1). At the beginning of the operation, the membrane resistance under the three working conditions showed a large increase immediately and then tended to be stable, which was similar to the results shown above. When the initial flux was 60 L/(m^2^·h), the membrane resistance rose the slowest and the final membrane resistance was the lowest. This result further showed that the CM was polluted quickly at the beginning, and under operating conditions with a critical flux, the membrane life cycle could be extended [65,66].

As for the combined process (Figure S2), in the early stage, the membrane resistance was only 4.5 × 10^11^ m^−1^, and with the increase in running time, the membrane resistance increased slowly. After 20 days, the membrane resistance increased to only 1.2 × 10^12^ m^−1^, which was far lower than the resistance found for direct filtration. This means that the membrane in the operation of the combined process had a better anti-pollution performance.

### 3.3. Membrane-Fouling Model

**Figure 8:**
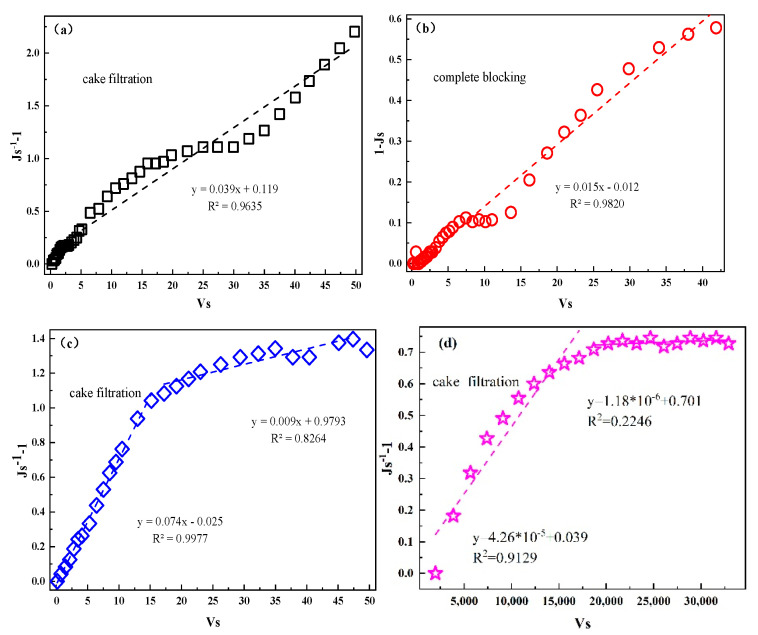
The membrane-fouling models with different initial fluxes were fitted with separate membrane filtration conditions: (**a**) the model with an initial flux of 30 L/(m^2^·h); (**b**) the model with an initial flux of 60 L/(m^2^·h); (**c**) the model with an initial flux of 90 L/(m^2^·h); (**d**) membrane-fouling model fitting for the combined process.

Membrane fouling is the main reason to restrict the operation of a membrane system, which can be further understood using model fitting. According to the conclusion above, different types of membrane fouling were created by different initial fluxes during the operating conditions when using direct filtration. The Hermia model was used to fit the model of membrane fouling and the result can be seen in Figure 8. The result showed that when the initial flux was 30, 60 and 90 L/(m^2^·h), the types of membrane fouling were more consistent with the models of cake filtration, complete blocking and cake filtration, respectively. This indicated that the cake layer on the membrane surface gradually formed in the stage of critical flux [67,68]. For the combined process, the result can be seen in Figure 8d. The result was more consistent with the cake layer model in the initial stage of operation, meaning that the cake layer was formed on the surface of the CM during this stage. This phenomenon might have been caused by the AC that was absorbed on the membrane surface because of the pumping action of the pump. In the subsequent stage of constant pressure filtration, although the cake layer had been formed, the fitting degree of the model of cake filtration was poor. It was speculated that the membrane fouling was dominated by various types of membrane fouling. This might have been because the small molecules that were created due to the decomposition of the pollutants in the wastewater by AOPs entered into the holes of the membrane.

## 4. Conclusions

In this study, a combined process involving an AOP, AC absorption and CM filtration for the treatment of refinery wastewater was systematically built. The treatment effect and membrane-fouling behavior of direct filtration and the combined process on refinery wastewater were compared and analyzed. According to the results, the following conclusions were found:

1. The TOC, COD and turbidity could be significantly improved using the combined process. This was because some organic macromolecular matter, such as aromatic compounds in the wastewater, was effectively decomposed using Fenton oxidationand finally removed using the AC and CM.
2. Compared with direct membrane filtration, it was found that the optimal critical flux of the CM could be significantly increased and the membrane fouling could be effectively alleviated using the combined process. According to the analysis, the AOP and AC absorption could decompose the macromolecular substances in the wastewater into small molecular substances, which passed through the pores of the membrane easier.
3. The model of membrane fouling in the combined process was more consistent with the cake layer model in the initial stage and the membrane fouling might have been dominated by various types of membrane fouling in the subsequent stages.

## Figures and Tables

**Table 1:** The equations of the Hermia model: *J*′s = *p*_0_/*p*, *p* represented the *TMP* data at a given time (kPa), *p*_0_ represented the initial *TMP* data when the filtration begins (kPa), Vs was the cumulative filtrate volume and *k*′ was the model constant.

Model	Equations	Model	Equations
Cake filtration	$\frac{1}{{J_{s}}^{\prime }}=1+k^{\prime }V_{s}$	Standard blockage	$J_{s}^{{}^{\prime }\frac{1}{2}}=1-\frac{k^{\prime }}{2}V_{s}$
Intermediate blockage	$\ln J_{S}^{\prime }=-k^{\prime }V_{s}$	Complete blockage	$J_{s}^{\prime }=1-k^{\prime }V_{s}$

## Data Availability

Not applicable.

## Supplementary Materials

The following are available online at https://www.mdpi.com/article/10.3390/membranes11090651/s1, Figure S1: The structure of the membrane module, Figure S2: Membrane resistance of CM in the operation of direct filtration and combined process: The membrane fouling resistance was calculated by Equations (1)–(4).
