# Supplementary material for: Adsorption-Enhanced Ceramic Membrane Filtration Using Fenton Oxidation for Advanced Treatment of Refinery Wastewater: Treatment Efficiency and Membrane-Fouling Control
Source: Membranes (Basel). 2021 Aug 25;11(9):651. doi: 10.3390/membranes11090651 (PMC8467550; doi:10.3390/membranes11090651)
Supplement: Supplementary file 1 [file membranes-11-00651-s001.zip › membranes-1291726-supplementary.pdf]

Fenton-Oxidation Adsorption-Enhanced Ceramic Membrane Filtration for Advanced Treatment of Refinery Wastewater: Treatment Efficiency and Membrane-Fouling Control

Haotian Mu[1], Qi Qiu[3], Renzhen Cheng[1], Liping Qiu[1,2,*], Kang Xie[1,2], Mingchang Gao[1] and Guicai Liu[1,2,*]

1 School of Civil Engineering and Architecture, University of Jinan, Jinan 250022, China; muhaotian1997@163.com (H.M.); 18366105822@163.com (R.C.); cea_xiek@ujn.edu.cn (K.X.); gaomc555@163.com (M.G.)

2 Research Center for Functional Material & Water Purification Engineering of Shandong Province, Jinan 250022, China

3 School of Water Conservancy and Environment, University of Jinan, Jinan 250022, China; qiuqi_1994@163.com

*Correspondence: lipingqiu@163.com (L.Q.); cea_liugc@ujn.edu.cn (G.L.)

Submitted to

Membranes

August 25, 2021

*To whom correspondence should be address.

Tel: 13793189608 e-mail: lipingqiu@163.com; Tel: 18769707072 e-mail: cea_liugc@ujn.edu.cn

List of Supplementary materials

Figure S1: The structure of the membrane module.

Figure S2: Membrane resistance of CM in the operation of direct filtration and combined process: The membrane fouling resistance was calculated by equation (1-4).

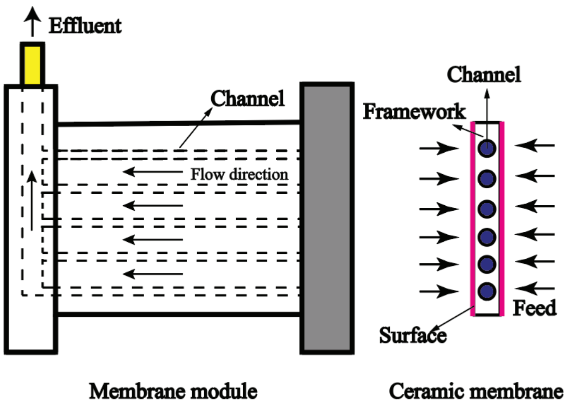

Figure S1: The structure of the membrane module.

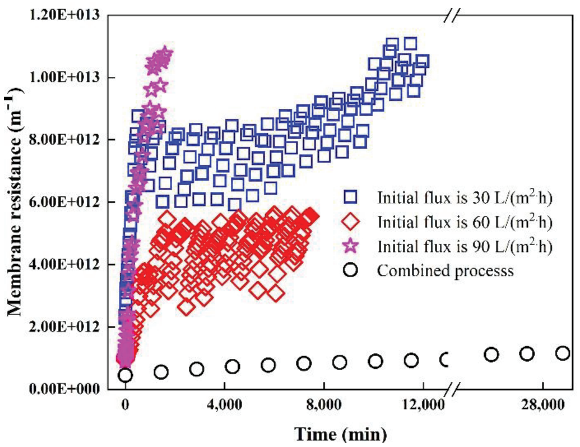

Figure S2: Membrane resistance of CM in the operation of direct filtration and combined process: The membrane fouling resistance was calculated by equation (1-4).
